# Durable Hematological and Major Cytogenetic Response in a Patient with Isolated 20q Deletion Myelodysplastic Syndrome Treated with Lenalidomide

**DOI:** 10.1155/2014/949515

**Published:** 2014-02-12

**Authors:** Bagi Jana, Anas Khanfar, Mary Ninan

**Affiliations:** ^1^The University of Texas Medical Branch, 301 University Boulevard, Galveston, TX 77555, USA; ^2^Department of Internal Medicine, 301 University Boulevard, Galveston, TX 77555, USA; ^3^Divisions of Hematology and Oncology, 301 University Boulevard, Galveston, TX 77555, USA

## Abstract

Myelodysplastic syndrome (MDS) is a clonal bone marrow disorder characterized by ineffective hematopoiesis. It is characterized by peripheral blood cytopenia and significant risk of progression to acute myeloid leukemia result. Deletion of the long arm of chromosome 20 (20q deletion) is present in 3–7% of patients with MDS. Lenalidomide is an immunomodulatory agent with antiangiogenic activity. It is FDA approved for the treatment of anemia in patients with low or int-1 risk MDS with chromosome 5q deletion with or without additional cytogenetic abnormalities. Study of lenalidomide in patients with MDS without 5q deletion but other karyotypic abnormalities demonstrated meaningful activity in transfusion dependent patients; however, response of patients with isolated 20q deletion to lenalidomide is not known. We are reporting a patient with 20q deletion MDS treated with lenalidomide after he failed to respond to azacytidine; to our knowledge this is the first report of a patient with isolated 20q deletion treated with lenalidomide.

## 1. Introduction

Myelodysplastic syndrome (MDS) is a clonal bone marrow disorder characterized by ineffective hematopoiesis. The FDA approved lenalidomide for the treatment of anemia in patients with low or int-1 risk MDS with chromosome 5q deletion with or without additional cytogenetic abnormalities.

Response of patients with isolated 20q deletion to lenalidomide is not known. We are reporting a patient with 20q deletion MDS treated with lenalidomide after he failed to respond to azacytidine; to our knowledge this is the first report of a patient with isolated 20q deletion treated with lenalidomide.

## 2. Case Report

**Figure 1:**
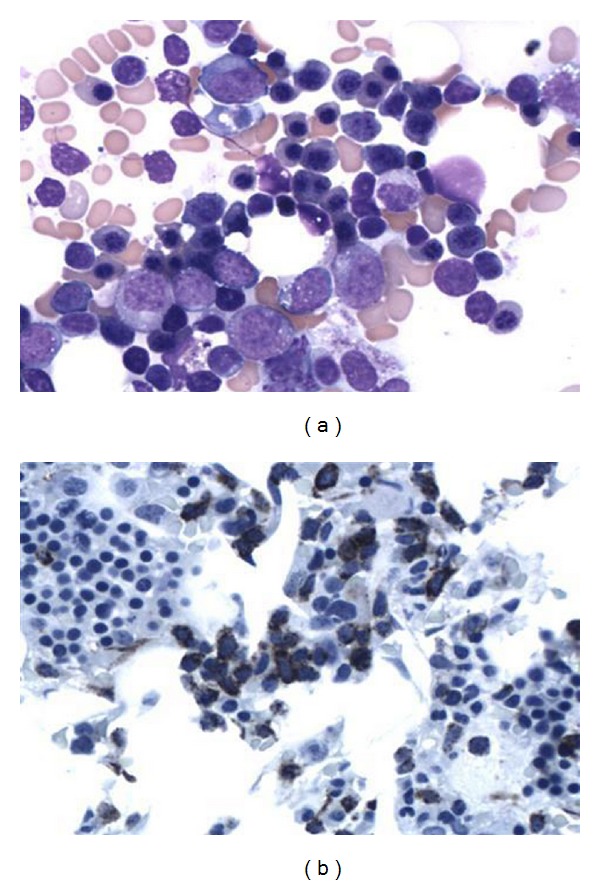
(a) Bone marrow aspirate on diagnosis showing myeloid hyperplasia with blasts. (b) Bone marrow aspirate at diagnosis with CD34 stain.

**Figure 2:**
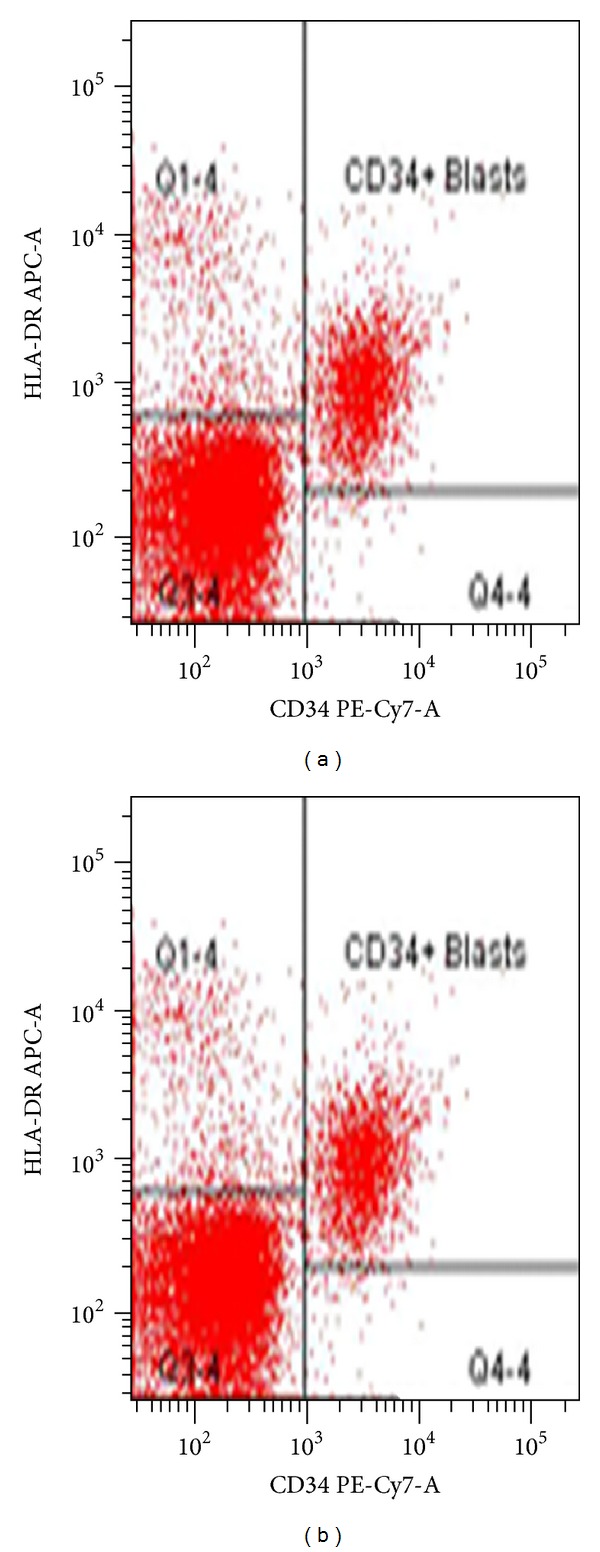
(a) Flow cytometry results at diagnosis with gating scheme for CD34 versus HLA-DR. (b) Flow cytometry results at diagnosis with gating scheme CD13 versus CD34.

**Figure 3:**
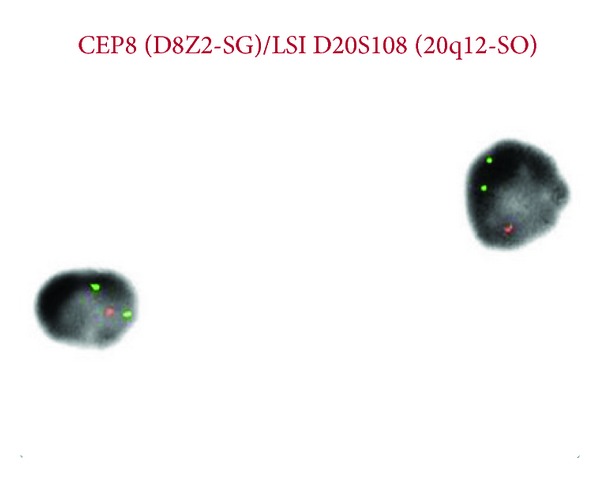
MDS FISH: chromosomes 8 and 20.

A 67-year-old man presented with feeling progressively weaker for few weeks. He noticed easy bruising several days prior to presentation. No overt bleeding or fever was reported. Patient was unable to function due to progressive weakness. Complete blood count revealed pancytopenia with WBC count of 1.6 M/mcL, hemoglobin of 8.6 g/dL, and platelet count of 53 K/mcL. absolute neutrophil count (ANC) was 600 K/mcL. MCV was 99.0 fL. Bone marrow aspirate and biopsy revealed increased myeloid blasts suggestive of high-grade myelodysplastic syndrome (as shown in Figures 1(a) and 1(b)). Flow cytometric analysis of bone marrow showed increased myeloid blasts expressing dim CD45, CD13, dim CD33, CD34, CD117, and HLA-DR (as shown in Figures 2(a) and 2(b)). Blasts represented 11% of marrow cellular events. IPSS cumulative score of 2 was determined by 11–20% of blasts (1.5) and 2-3 cytopenias (0.5). MDS and MLL FISH revealed abnormal signal pattern with 20q deletion. FISH analysis with specific probes (5q31, 7q31, 20q12, centromere 8, and 11q23) revealed deletion of 20q12 locus in 32.5% (65/200) of nuclei examined (as shown in Figure 3). Results from all other probes were within normal limits. Cytogenetic analysis revealed normal male karyotype. A 20q-metaphase was not seen despite additional cell analyses suggesting that 20q-clone, while seen by interphase cells by FISH, may not be mitotically active. Cytogenetic and karyotype testing were performed in UCLA, Dept. of Pathology and Laboratory Medicine, and Genoptix Medical Laboratory performed flow cytometry. Final diagnosis was high-grade myelodysplastic syndrome consistent with refractory anemia with excess blasts.

Patient was started on azacytidine at 75 mg/m^2^ D1–D5 every 28 days. He tolerated the therapy well and did not have any undue nonhematological toxicity. He continued to have persistent cytopenia during the entire therapy and required blood product transfusions on a regular basis. Repeat bone marrow aspirate and biopsy after the 3rd cycle revealed persistent blasts constituting 21% of the marrow cellularity. After completing 6 cycles of azacytidine he underwent repeat bone marrow aspirate and biopsy which revealed persistent marrow abnormalities with 10% myeloblasts and 20q12 (deletion 20q) in 10% of the nuclei examined. Patient continued to experience significant cytopenia.

Patient refused consideration of allogeneic bone marrow transplantation. He did not wish to consider conventional chemotherapy. Given the progressive cytopenia, persistent increased marrow blasts, need for blood product transfusions, and success of lenalidomide in the treatment of 5q deletion and efficacy in reducing transfusion requirement in the non-5q deletion, a novel treatment approach with lenalidomide 10 mg po daily was recommended.

**Figure 4:**
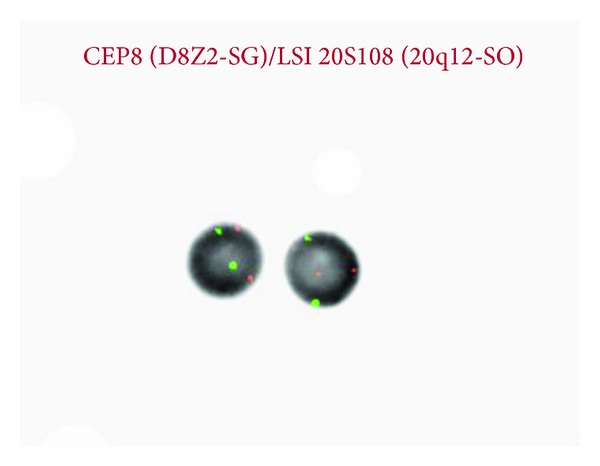
FISH MDS. Chromosomes 8 and 20 after 3 weeks of lenalidomide treatment.

Patient tolerated lenalidomide very well and experienced grade 1 facial rash toxicity. Three weeks after starting the therapy, his WBC counts improved to 3.4 k/uL, hemoglobin improved to 12.7 g/dL, and platelets increased to 102 k/uL. He had a normal differential and had no circulating immature cells. Repeat bone marrow aspirate and biopsy revealed evidence of normal hematopoiesis and no morphological evidence for MDS that was present in the previous 3 biopsies. Flow cytometry revealed 97% viable cells with CD 34-positive blasts estimated at 3% (as shown in Table 1). MDS FISH with the same panel of probes done in the previous biopsies revealed absence of 20q deletion (as shown in Figure 4).

Clinically patient felt much better and maintained the improved blood counts. Patient remained in complete hematologic remission 8 months after initiating the therapy. He did not require any further blood product transfusions during this period. He continued lenalidomide without any major complications. He returned to full time duties. After the 8-month period, he was noted to have recurrent cytopenia. He underwent repeat bone marrow biopsy, which indicated relapse with presence of 20q deletion. He continued to refuse bone marrow transplantation and he was enrolled in a clinical trial that is ongoing.

## 3. Discussion

MDS is characterized by peripheral blood cytopenia and significant risk of progression to acute myeloid leukemia result (AML) [1]. Deletion of the long arm of chromosome 20 (20q deletion) is present in 3–7% of patients with MDS. 20q deletion can be present along with other cytogenetic abnormalities or as an isolated defect [2–6]. MDS with isolated 20q deletion accounted for 1.3–2% in various large series of patients with MDS [4–7].

Case series published by Braun et al. consisting of patients with MDS with isolated 20q deletion revealed that 43.5% had refractory anemia, according to 2001 WHO criteria [8]. Patients with isolated 20q deletion had significantly reduced marrow percentage of blasts, lower platelet count, and higher reticulocyte count when compared with clinical features of patients without 20q deletion. Median survival of patients with isolated 20q deletion was 54 months. Overall, MDS in patients with isolated 20q deletion was associated with features of low risk and favorable prognosis [8]; nevertheless patients with 20q deletion are treated similarly to patients with non-5q deletion MDS. Existing therapies are not always curative and more treatment options are needed.

Lenalidomide is an immunomodulatory agent with antiangiogenic activity [9, 10]. A study of lenalidomide in patients with low or int-1 risk MDS associated with chromosome 5q interstitial deletion either alone or with additional chromosomal abnormalities revealed meaningful increase in hemoglobin, RBC transfusion independence, and cytogenetic response [11].

Study of lenalidomide in patients with MDS without 5q deletion but other karyotypic abnormalities demonstrated meaningful activity in transfusion dependent patients. In this same study, authors concluded that clonal suppression was uncommon and histological remission rare [12]. There was no patient with isolated 20q deletion included in this study although there was one patient with deletion 20q with add 20p, on review of the various karyotypic abnormalities.

Isolated 20q deletion is frequently reported in MDS. Approach to this group of patients is similar to non-5q deletion MDS. To our knowledge, activity of lenalidomide in this group of patients is neither reported nor known. In our patient, lenalidomide therapy was initiated as a novel attempt to help treat his MDS especially since he previously failed the standard of care azacytidine and continued to refuse other viable options including bone marrow transplantation.

This patient presented with severe symptomatic cytopenia due to MDS with IPSS score of 2. He did not have significant hematological response to azacytidine over several months. Bone marrow aspirate and biopsy done on various different occasions, before and during azacytidine, revealed isolated 20q deletion. He continued to have morphological evidence of MDS in the marrow. Patient had persistent high percentage of blast in the bone marrow on the three bone marrow biopsies ranging from 10 to 21. He declined to consider allogeneic bone marrow transplantation. He had a surprisingly brisk response to lenalidomide and achieved completed histological and cytogenetic response three weeks after starting the therapy.

Lenalidomide is an active therapy in patients with 5q deletion; it produces meaningful hematological response and reduces need for RBC transfusion and cytogenetic response [11]. MDS-003 studies indicate that lenalidomide mechanism of action in MDS is karyotype dependent. MDS-003 investigators reported 45% complete cytogenetic response in patients with 5q deletion syndrome with lenalidomide and bone marrow morphological features returned to normal in 36% of patients. Onset of response to lenalidomide was brisk, usually within 4–8 weeks.

In contrast Raza et al. reported that, in non-5q deletion MDS population, lenalidomide produced significant erythroid response without producing complete cytogenetic response or histological remission [12]. It is notable that Raza et al's. study did not include any patient with an isolated 20q deletion; one patient with 20q deletion with add 20p marker was included and that patient experienced cytogenetic progression. In another case report from China, Xu et al. reported a patient with combined 5q and 20q deletion. When treated with lenalidomide, complete erythroid response occurred and 5q deletion clone was suppressed while 20q deletion clone expanded [13]. Neither of these two patients had isolated 20q deletion.

In our patient, we noted complete cytogenetic response along with complete hematological and histological remission three weeks after starting lenalidomide. These findings are thought provoking and raise the possibility that lenalidomide is active in patients with isolated 20q deletion MDS and can act in a manner different than when it cooccurs with other cytogenetic abnormalities. Up to 2% of patients with MDS have isolated 20q deletion syndrome. Further studies to better elucidate the mechanism of action, efficacy, and role of lenalidomide in patients with isolated 20q MDS deletion are needed.

## Figures and Tables

**Table 1:** Flow cytometry after 3 weeks of lenalidomide treatment.

Flow cytometry differential (% of total cells)	
Lymphocytes	44
B-cells	4
Kappa	1
Lambda	<1
Kappa : Lambda ratio	2
T-cells	39
CD4	28
CD8	11
CD4 : CD8 ratio	2.7
CD3+CD56+	2
Natural killer cells	1
Monocytes	5
Granulocytes	42
CD34-positive blasts	3
Plasma cells	<1
Viability	97
